# Update on human exposure to glyphosate, with a complete review of exposure in children

**DOI:** 10.1186/s12940-020-00673-z

**Published:** 2020-11-12

**Authors:** Christina Gillezeau, Wil Lieberman-Cribbin, Emanuela Taioli

**Affiliations:** The Institute for Translational Epidemiology at the Icahn School of Medicine at Mount Sinai, One Gustave L Levy Place, Box 1133, New York, NY 10029 USA

**Keywords:** Review, Round up, Youth, Glyphosate exposure, AMPA, Urinary biomarkers

## Abstract

**Background:**

Glyphosate, a commonly used pesticide, has been the topic of much debate. The effects of exposure to glyphosate remains a contentious topic. This paper provides an update to the existing literature regarding levels of glyphosate exposure in occupationally exposed individuals and focuses or reviewing all the available published literature regarding glyphosate exposure levels in children.

**Methods:**

A literature review was conducted and any articles reporting quantifiable exposure levels in humans published since January 2019 (the last published review on glyphosate exposure) were reviewed and data extracted and standardized.

**Results:**

A total of five new studies reporting exposure levels in humans were found including 578 subjects. Two of these studies focused on occupationally exposed individuals while three of them focused on glyphosate exposure levels in children. Given the sparse nature of the new data, previously identified studies on exposure to glyphosate in children were included in our analysis of children’s exposure. The lowest average level of glyphosate exposure reported was 0.28 μg/L and the highest average exposure levels reported was 4.04 μg/L.

**Conclusion:**

The literature on glyphosate exposure levels, especially in children, remains limited. Without more data collected in a standardized way, parsing out the potential relationship between glyphosate exposure and disease will not be possible.

## Introduction

The concerns associated with exposure to glyphosate, the active ingredient in the pesticide Round Up, have been a topic of much debate due to recent rulings and legal cases in the United States against the company that manufactures Round Up, Monsanto, which have concluded that the chemical may be carcinogenic [1]. Despite its widespread use worldwide and because of the introduction of genetically modified seeds that are Round-Up resistant, and its adoption in the early 2000s as a mean to speed up crop desiccation [2], the amount of exposure in the general population and the potential effects of sustained exposure on human health are largely unknown. The amount of permissible residues of glyphosate on crops has increased correspondingly [3], despite the fact that the literature on the health effects is scarce. A recent meta-analysis has suggested that glyphosate use in an occupational setting may raise the risk of non-Hodgkin lymphoma as much as 41% [4]; a pooled analysis of case-control studies from North America confirmed the association, and suggested that specific histologic subtypes of non-Hodgkin lymphoma may be associated with exposure to glyphosate [5]. As with any chemical, there are multiple steps involved in evaluating risk, which include gathering information about human exposures, so that the levels that produce harm in one population or animal species can be compared to typical exposure levels. However, we have previously shown that data on human exposure in workers and the general population are very limited [6]. Several other gaps in knowledge exist around this product, for example results on its genotoxicity in humans are limited. The continued debate regarding the effects of glyphosate exposure makes establishing exposure levels in the general public a pressing public health issue, especially for the most vulnerable. As such, we have endeavored to summarize what is known about exposure levels of glyphosate and its metabolite, Aminomethylphosphonic acid (AMPA). Research shows that children are especially vulnerable to environmental toxins in general due to their small body mass; in the case of glyphosate, they are also more likely to be exposed due to contact with dirt in playgrounds [7]. As a result, children and occupationally exposed adults are the individuals most likely to experience harm from glyphosate exposure. We are now presenting an updated review of the literature on exposure to glyphosate, with a focus on children’s exposure.

## Methods

We conducted a PubMed and Google Scholar search using the following terms: “glyphosate” (“glyphosate” OR “1071-83-6” OR “roundup” OR “N-(Phosphonomethyl)glycine”) or (((“AMPA”) NOT “AMPA receptor”)) OR “Aminomethylphosphonic acid”) AND (“human”) from November 12,018, the date of our last literature review, to March 312,020. No limitation on language of the publication was imposed on the search. The search returned 181 results. After title and abstract review, 147 studies were excluded because they did not report information on exposure levels in human subjects. After a full article review, a further 29 studies were excluded because they did not report a quantifiable level of exposure for their participants. Five studies were ultimately included in this updated review, which included two studies that focused on urinary glyphosate levels in children and three studies that focused on urinary glyphosate levels in occupationally exposed adults. Because of the very limited data available regarding exposure levels in children, data from two previously identified studies were also included in order to report a complete, updated analysis on exposure in children [8, 9].

Average glyphosate levels were abstracted and standardized to μg/L. For the three studies where the geometric mean (GM) was not reported, the GM was estimated [10] from the arithmetic mean (AM) using the formula $GM=\frac{AM^2}{\sqrt{AM^2+{SD}_y^2}}$, where SD_x_ is the standard deviation of the data on the native scale and AM is the arithmetic mean of the data on the native scale. For the two studies where the range was not reported, it was estimated to be 3SD_y,_ as this would account for 99.7% of the values.

## Results

There were five new studies published in the past 2 years and included in this update, two of which measured urinary glyphosate levels in children [11, 12], and three [13–15] measured urinary glyphosate levels in agricultural workers. One of those three studies also provided urinary glyphosate levels in non-agricultural workers as a comparison group [15]. A total of 578 new individuals were included among all of the studies with 389 children, 38 adults in the general population and 151 adult agricultural workers. The lowest LOD for glyphosate reported was 0.01 μg/L [12] and the highest was 5 μg/L [14]. The lowest average level of urinary glyphosate within the detectable results reported was 0.28 μg/L [10] and the highest was 4.04 μg/L [13]. Only two of the studies reported AMPA levels in the included subjects [13, 14] (Table 1).

**Table 1:** Description of the new studies identified and included in this review

Citation number, Author, year	Country	Year of sampling	Subjects	Number of subjects	Lab methods	Type of sample	Creatinine Adjusted levels	LOD glyphosate	LOD AMPA	Samples below LOD included in average	Glyphosate Results	AMPA Results
**OCCUPATIONAL EXPOSURE**												
Perry, 2019 [13]	US (Wisconsin)	1997–1998	Farmers who self-reported glyphosate exposure 8 h prior to sample collection and farmers who did not report glyphosate use	18 farmers with glyphosate use, 17 farmers without glyphosate use	LC-MS/MS	Urine	No	0.4 μg/L	1 μg/L	No	Farmers using G: 39% had detectable levels, mean (range):4.04 μg/L (1.3–12.0 μg/L). Farmers not using G: None had detectable levels	Only one G using farmer with the highest G level had detectable AMPA at 4.1 μg/L
Balderrama-Carmona 2019^a^ [14]	Mexico (Valle del Mayo)	NR	Farmers who lived in small communities in Mexico and reported regular application of pesticides	30 urine samples from agricultural workers	HPLC	Urine	Not specified	5 μg/L	15 μg/L	NA	Of the 30 samples tested, none of them had detectable levels of glyphosate	Of the 30 samples tested, 6% had detectable levels of AMPA of 0.42 and 2.23 μg/L
Wongta, 2018 [15]	Thailand	2017	Rice, Longan, and Vegetable farmers living in San Pa Tong District	38 Rice Farmers, 31 Longan Farmers, 17 Vegetable Farmers	FMOC-Cl prior to HPLC	Urine	Not specified for G	0.5 μg/L	NR	Not specified	Rice: G was detected in 10.5% of samples Mean ± SD: 2.01 ± 0.81 μg/L GM: 1.89 μg/L; Longan: G detected in 30% of samples, Mean ± SD 2.88 ± 1.46 μg/L, GM: 2.55 μg/L; G was detected in 23.5% samples, Mean ± SD: 3.11 ± 1.15 μg/L, GM: 2.92 μg/L	NR
**TOTAL (*n*** **= 3)**				**151**								
**GENERAL POPULATION**												
Wongta, 2018 [15]	Thailand	2017	Nonfarm workers between 18 and 65 living in San Pa Tong District in Thailand	38	FMOC-Cl prior to HPLC	Urine	Not specified for G	0.5 μg/L	NR	Not specified	G was not detected in the urine of any non-farm workers	NR
**TOTAL (*n*** **= 1)**				38								
**CHILDREN**												
Trasande 2020 [11]	US (Seattle, WA, New York, NY)		Bright Start: Neonates younger than 30 days Early Start: infants 10–19 months PEEPS: children ages 3–8 years	Bright Start: 10 Early Start: 66 PEEPS: 32 Total of 108	HPLC-MS/MS	Urine	No	0.11	NR	Yes, values below the LOD imputed as LOD/sqrt(2)	The majority of the samples were below the LOD and the mean (SD) glyphosate concentration was 0.28 (0.23)	NR
Sierra-Diaz, 2019 [12]	Mexico	NR	Children under the age of 17 living in Agua Caliente Children under the age of 12 living in Ahuacapán	192 Agua Caliente; 89 Ahuacapán	HPLC/MS/MS	Urine	Not specified	Curve range: 0.01–1000 μg/L	NR	Not specified	Agua Caliente: G was detected in 72.91% of samples with a mean ± SD of 0.363 ± 0.321 μg/L Ahuacapán: G was detected in 100% of the samples with a mean ± SD: 0.606 ± 0.5435 μg/L	NR
**TOTAL (*n*** **= 2)**				**389**								

^a^Values reported in this study do not match the reported LOD. Authors were contacted to clarify but did not respond

### Studies on children

In a study from the United States, urinary glyphosate levels were measured using HPLC-MS/MS (LOD 0.1 μg/L and LOQ 0.33 μg/L) in urine samples from children in three different age ranges. Ten newborns (less than 30 days old), 66 infants (10–19 months of age), and 32 children (ages 3 to 8 years), provided urine samples to measure glyphosate exposure levels in New York, NY and Seattle, WA within the United States. The AM± SD glyphosate level was calculated including all 108 children with values below the LOD imputed as $\frac{LOD}{\sqrt{2}}$ and was equal to 0.28 ± 0.29 μg/L; the range of detectable values was 0.105–2.125 μg/L. The mean ± SD levels were not reported by age group, although the median urinary glyphosate levels were below the LOD in each age group [11].

Urinary levels of glyphosate were measured using HPLC-MS/MS (LOD: 0.01–1000 μg/L) in 192 children < 17 years living in Agua Caliente and 89 children < 12 years living in Ahuacapán, two rural areas in Mexico; 72.91% of the children in Agua Caliente had detectable levels of glyphosate (AM ±SD: 0.363 ± 0.321 μg/L), versus 100% in Ahuacapán (AM± SD: 0.606 ± 0.5435 μg/L) [12].

We previously identified two studies that reported glyphosate levels in children, one of which was conducted in the US, while the other was conducted in Denmark. The first study was conducted in Iowa in 2007; urinary glyphosate levels were measured in 182 samples from 51 children from non-farming households and in 235 samples from 66 children from farming households, using a fluorescent covalent microbead immunoassay (LOD: 0.09 μg/L). Values below the LOD were included as 0, and all values were log-transformed prior to modeling. Excluding the 66 samples (22 from non-farming households and 44 from farming households) with levels reported as non-detected or below the LOD, the range of glyphosate levels was 0.10–9.4 μg/L for non-farming children and 0.022–18 μg/L for farming children. Glyphosate values were above the LOD in 88% of the children from non-farming, and 81% of the children from farming households (adjusted geometric means: 2.5 (95% CI: 2.1–3.1) μg/L and 1.9 (95% CI: 1.3–2.5) μg/L respectively). Values in children were higher than levels of their parents, regardless of whether the parents worked on a farm or not [8].

**Fig. 1:**
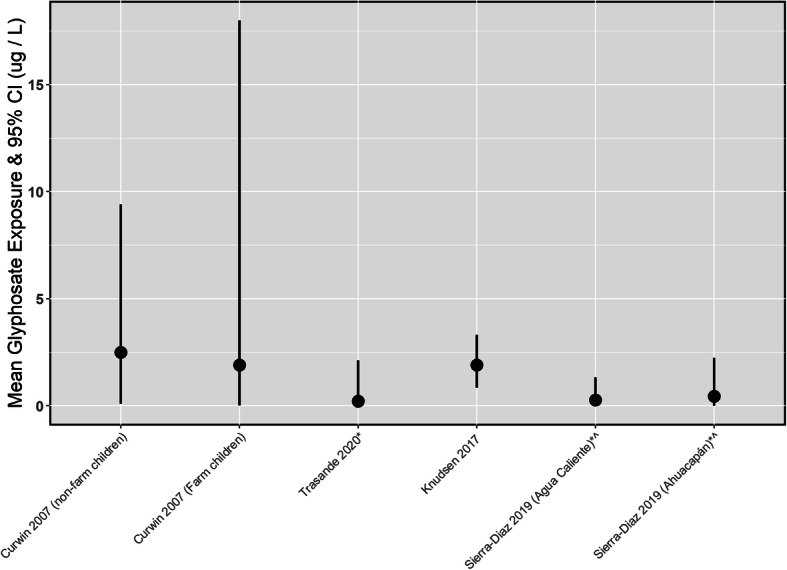
Urinary Geometric Mean Glyphosate Concentrations in Children. *GM was estimated from AM and SD; ^^^Range was estimated from SD; ^~^SD was estimated from range and AM

In the Danish study, urinary glyphosate levels of 14 children aged 6 to 11 years from both urban and rural communities were measured with ELISA (LOD: 2.5 μg/L). The AM level of glyphosate was 1.96 (range: 0.85–3.31) μg/L, and all of the samples had a detectable level, without significant differences between children from urban vs rural homes [9]. Because of the differing methods of analysis, no attempt at meta-analysis was completed on the studies that included children, but the average glyphosate levels reported in children ranged from 0.28 μg/L [6] to 2.5 μg/L [9] (Fig. 1).

#### Occupationally exposed individuals

In Perry 2019, urine samples were collected from 18 farmers who reported using glyphosate and 200 farmers who did not; samples were cryopreserved after being collected between 1997 and 1998 [13]. The urine samples from the 18 glyphosate users and 18 randomly selected samples from the pool of 200 glyphosate non-users were analyzed using high performance liquid chromatography tandem mass spectrometry (HPLC-MS/MS). The LOD for glyphosate was 0.4 μg/L and was 1 μg/L for aminomethylphosphonic acid (AMPA). One of the 18 glyphosate non-user samples could not be analyzed. Of the remaining 17 samples, none had glyphosate levels above the LOD.. In the 18 glyphosate user samples, seven of the 18 samples had detectable levels of glyphosate with a range of 1.3–12.0 μg/L and an AM of 4.04 μg/L. Only the sample with the highest level of glyphosate, 12 μg/L, had a detectable level of AMPA.

In a 2019 study by Balderrama-Carmona et al. from Valle del Mayo, Sonora Mexico, urine samples from 30 agricultural workers who applied herbicides were analyzed for glyphosate and AMPA using high-pressure liquid chromatography to calculate calibration curves using blanks and different concentrations of aqueous patterns of glyphosate and AMPA [14]. The correlation coefficient for the calibration curve for both AMPA and glyphosate was R^2^ = 0.994. The LOD for 5 μg/L for glyphosate and 15 μg/L for AMPA. In testing, 6% of samples had detectable levels of AMPA and none of the samples had detectable levels of glyphosate. The AMPA concentrations reported for the two urine samples that contained detectable levels of AMPA were 0.42 and 2.23 μg/L. Because of the discrepancy between the LOD and the actual urinary values reported, the study was considered of low quality.

**Fig. 2:**
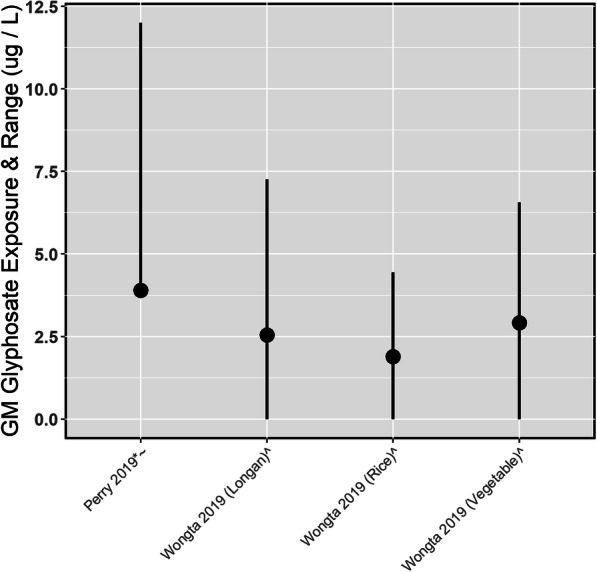
Urinary Geometric Mean Glyphosate Concentrations in Occupationally Exposed Adults. *GM was estimated from AM and SD; ^^^Range was estimated from SD; ^~^SD was estimated from range and AM

In a 2018 Thai study glyphosate levels in urine samples from 38 rice farmers, 31 longan farmers, and 17 vegetable farmers were measured using fluorenylmethyloxycarbonyl chloride prior to HPLC, which had an 80% recovery, an interbatch residual standard deviation of 3.0% and an intrabatch residual standard deviation of 8.9% [15]. The LOD was 0.5 μg/L. Ten percent of the rice farmers, 30% of the longan farmers, and 23% of the vegetable farmers had detectable levels of glyphosate. Among the rice farmers with detectable levels of glyphosate, the AM±SD glyphosate level was 2.01 ± 0.8 μg/L, among longan farmers was 2.88 ± 1.46 μg/L and among vegetable farmers was 3.11 ± 1.15 μg/L (Fig. 2).

## Discussion

The literature review shows that the amount of work published on glyphosate levels in the population continues to be very limited. In the last 2 years, only five new studies reporting actual values of glyphosate in humans were published, and even when combined with the studies in our previous review, only 4299 individuals have been tested worldwide [6, 8, 9, 11–15] for their urinary glyphosate level and only 520 of them are children [8, 9, 11, 12]. Only two of the new studies were conducted in the United States [11, 13], bringing the total number of studies conducted in the US to six. Another remarkable aspect of this review is the paucity of measurements of AMPA together with glyphosate. Measuring residues and metabolites allow to better understand the individual ability to degrade the main compound, as well as to detect other by-products, such as AMPA, which carries its own safety concerns [16]. Additionally, since glyphosate is non-persistent with an estimated biological half-life in urine ranging between five and a half and 10 h depending on the measurement and adjustment methods [17], measurement of AMPA levels within these samples is essential for accurate estimation of true exposure levels.

In terms of laboratory methods, fluorescent covalent microbead immunoassay [8], ELISA [9], HPLC [14], LC-MS/MS [13], HPLC-MS/MS [11, 12],and 9-fluorenylmethyloxycarbonyl chloride to derivatize urine samples prior to using high pressure liquid chromatography were deployed [15]. Although there is not a definitive best practice for measurement of glyphosate levels. Research with other non-persistent chemicals with short half-lives like bisphenol A has found that ELISA lacks the necessary sensitivity and specificity to accurately measure exposure [18]. MS provides a low LOD and is routinely used by the CDC’s Environmental Health Laboratory [19], suggesting it is likely a more robust measurement method.

When reviewing the published literature, we endeavored to find statistical methods for measuring the accuracy or at least consistency of the results reported. Most of the studies, however, did not report repeated measures that would allow for calculation of coefficient of variation or comparison across methods. Researchers might consider reporting the coefficient of variation or intra-correlation coefficient within the results to allow for better comparison between studies.

Of interest, there are now six data sets from four distinct studies reporting on children. All the studies confirm the presence of glyphosate in urine samples from children, both within and outside of agricultural communities, with values exceeding those measured in adults when the corresponding values were available. Previous research suggests multifactorial reasons for higher levels of environmental toxin exposure in children, which may include smaller body mass or higher likelihood of ingestion, particularly in younger children who are inclined to put non-food items in their mouths. Children may also be exposed through their play on the ground and in the dirt, particularly dirt from playgrounds, which may be contaminated by toxins deposited in the soil or dust. This may be particularly true in households where parents are occupationally exposed to toxins and may bring those toxins home inadvertently on their clothing, thereby increasing their child’s exposure. Additionally, even if children’s exposure levels are not higher than adults, their growth and maturation may be impacted by toxins in a way that adults’ will not be. Furthermore, they may be more likely to develop disease from exposure given the increased number of years for chronic exposure a child might have as compared to someone exposed in adulthood [7]. Given the evidence that children are especially vulnerable to environmental carcinogens [20, 21], tracking exposure to products such as glyphosate in children is a pressing public health priority. The lack of data on glyphosate exposure in children, and on time trends, geographic variability, and sources of exposure call for systematic monitoring of glyphosate and for more studies on the biological effects of the exposure in the general population as well as in vulnerable subsets.

The levels of glyphosate exposure in occupationally exposed individuals is also a major concern. Although the highest average urinary level of glyphosate reported in these updated studies (AM [range]: 4.0 [1.3–12.0] μg/L) [13] did not reach the highest level seen in previous studies we reviewed, (AM [range]:73.6 [40.2- > 80.0] μg/L) [22], the average urinary glyphosate level in occupationally exposed individuals is still disconcertingly high. The decrease in average levels may reflect better personal protective equipment usage, in light of the increased publicity surrounding the potential hazards of glyphosate exposure. However, given the results of a 2017 study showing glyphosate and AMPA levels increasing over time in non-farmer US and European adults [23], it the current result is likely a reflection of a smaller number of studies, only one of which was conducted in the US, published in the last few years compared to the last review we published.

In terms of methodological aspects, of the three studies that measured occupational exposure, two collected convenience samples from farmers, rather than collecting samples at set intervals before and after exposure. None of the studies collected information on urinary glyphosate levels prior to occupational use. This, combined with the lack of data on glyphosate levels in the non-occupationally exposed general population, makes parsing out the various sources of the observed glyphosate levels in the occupationally exposed difficult, as such levels can also derive from food, drinking water, wind or dust in addition to occupational exposure [24–26]. We suggest that studies measuring glyphosate levels in occupationally exposed individuals should attempt to collect samples prior to application, the day of application, and in the days after application, as well as gathering information about how regularly farmers apply glyphosate. This will provide sufficient information to distinguish a baseline level of exposure from the effect of recent application. Regardless, without more standardized measurement practices, it remains difficult to establish just how much more exposure occupationally exposed individuals have compared to the average adult.

Monitoring of urinary glyphosate levels should be conducted in the general population, but is especially important for those who are occupationally exposed and those vulnerable. We continue to suggest that inclusion of glyphosate as a measured exposure in nationally representative studies like the National Health and Nutrition Examination Survey will allow for a better understanding of the risks that glyphosate may pose and allow for better monitoring of those who are most likely to be exposed and those who are more susceptible to the exposure.

## Data Availability

This is a review and all data is publicly available.

## Abbreviations

LOD: Limit of Detection
ELISA: enzyme-linked immunosorbent assay
PPB: Parts per billion
HPLC: High-Performance Liquid Chromatography
MS/MS: Tandem Mass Spectrometry
AMPA: Aminomethylphosphonic Acid
